# Long-Term Outcomes and Prognostic Factors of Trabeculectomy following Intraocular Bevacizumab Injection for Neovascular Glaucoma

**DOI:** 10.1371/journal.pone.0135766

**Published:** 2015-08-14

**Authors:** Tomomi Higashide, Shinji Ohkubo, Kazuhisa Sugiyama

**Affiliations:** Department of Ophthalmology and Visual Science, Kanazawa University Graduate School of Medical Science, Kanazawa, Japan; University of Illinois at Chicago, UNITED STATES

## Abstract

**Purpose:**

To evaluate long-term outcomes and identify prognostic factors of trabeculectomy following intraocular bevacizumab injection for neovascular glaucoma.

**Methods:**

Sixty-one eyes of 54 patients with neovascular glaucoma treated by trabeculectomy following intraocular bevacizumab injection were consecutively enrolled. Surgical success criteria were sufficient intraocular pressure (IOP) reduction (IOP ≤21 mmHg, ≥20% IOP reduction, no additional medications or glaucoma surgeries) without devastating complications (loss of light perception, phthisis bulbi, and endophthalmitis) or significant hypotony (IOP ≤5 mmHg continued ≥6 months and until the last follow-up visit or hypotony requiring intervention). Kaplan-Meier survival curves and Cox regression analysis were used to examine success rates and risk factors for surgical outcomes.

**Results:**

The follow-up period after trabeculectomy was 45.0 ± 22.2 months (mean ± standard deviation). Surgical success rate was 86.9 ± 4.3% (± standard error), 74.0 ± 6.1%, and 51.3 ± 8.6% at 1, 3, and 5 years. Multivariate Cox regression analysis identified two risk factors; lower preoperative IOP (≤30 mmHg) for surgical failure and hypotony [hazard ratio (HR), 2.92, 6.64; 95% confidence interval (CI), 1.22 to 7.03, 1.47 to 30.0; P = 0.018, 0.014, respectively], and vitrectomy after trabeculectomy for surgical failure with or without hypotony criteria (HR, 2.32, 4.06; 95% CI, 1.02 to 5.28, 1.30 to 12.7; P = 0.045, 0.016, respectively).

**Conclusions:**

The long-term outcomes of trabeculectomy following intraocular bevacizumab injection for neovascular glaucoma were favorable. Lower baseline IOP was associated with development of significant hypotony, while additional vitrectomy was related to insufficient IOP reduction.

## Introduction

Neovascular glaucoma (NVG) is a refractory type of secondary glaucoma caused by neovascularization which occludes the trabecular meshwork. Many cases are not controlled medically and require surgical intervention [1]. Surgical outcome of trabeculectomy is often unfavorable, even with the use of antimetabolites, due to hemorrhagic complications or marked inflammation that stem from active neovascularization [2,3]. Risk factors for surgical failure of trabeculectomy in eyes with NVG include younger age [2,4], previous pars plana vitrectomy (PPV) [3,4], extensive peripheral anterior synechia [3], pseudophakia [5] and postoperative hyphema [6].

Almost two decades ago, vascular endothelial growth factor (VEGF), derived from ischemic retinal pathologies, was identified as the main causative molecule for anterior segment neovascularization in NVG [7,8], and intraocular injection of anti-VEGF agents has been proposed as a promising therapy for NVG. Since the anti-VEGF cancer drug bevacizumab became available for off-label use, it has been increasingly used to treat NVG. The intraocular injection of bevacizumab prior to trabeculectomy suppressed postoperative hyphema [9–11] and, in some studies, improved short-term intraocular pressure (IOP) outcomes of trabeculectomy [9,12]. Thus, although tube shunt surgeries have gained popularity as the first choice surgery in complex cases including NVG, trabeculectomy with adjunctive anti-VEGF injections may also work well for NVG.

However, the effects of intraocular anti-VEGF injections on the long-term surgical success and on the risk factors for surgical failure of trabeculectomy remain to be elucidated. Regarding the surgical success criteria, the guidelines of the World Glaucoma Association recommend setting a lower IOP limit (i.e. 6 mmHg) for IOP success [13], but many previous reports did not set the lower limit. This may make the comparison of surgical success between different studies complicated. The aim of this study was to evaluate long-term outcomes and to identify prognostic factors of trabeculectomy following intraocular bevacizumab injections for NVG. In addition, the effects of setting the lower IOP limit on the surgical success and the prognostic factors were also investigated.

## Materials and Methods

### Study design

The retrospective chart review of consecutive NVG cases treated by trabeculectomy with adjunctive intraocular injection of bevacizumab was approved by the Institutional Review Broad (IRB) of Kanazawa University Graduate School of Medical Science. The off-label use of bevacizumab (Avastin; Roche Pharmaceuticals, Basel, Switzerland) was also approved by the IRB. The study was performed in accordance with the tenets of the Declaration of Helsinki. Written informed consent about the glaucoma treatment was obtained from all patients after thorough discussion of the potential benefits and risks of intraocular bevacizumab injection. All patients were treated at Kanazawa University Hospital from 2007 through 2014. NVG was diagnosed by the presence of active neovascularization in the iris and/or angle, high IOP (≥21 mmHg) and underlying ischemic retinal diseases. The data of both eyes were collected separately in bilateral cases. A bevacizumab injection >4 months prior to trabeculectomy was not regarded as ‘adjunctive’ and was excluded since recurrence of anterior segment neovascularization after a bevacizumab injection in NVG eyes was likely to occur within 4 months [14, 15]. Eyes with short follow-up periods (<12 months) after trabeculectomy were also excluded.

### Strategies of NVG treatment

Our indication for intraocular bevacizumab injections was presence of active anterior segment neovascularization and visual acuity better than light perception with either high IOP (≥21 mmHg) under maximum medical therapy or opaque media preventing completion of transpupillary panretinal photocoagulation (PRP) [14]. Trabeculectomy was combined with PPV, cataract surgery, or both when completion of transpupillary PRP was not expected to be possible after surgery. Transpupillary PRP was added pre- and/or post-operatively when it was regarded as insufficient. Intraocular bevacizumab injections were performed as described previously [9]. Briefly, after sterilization and local anesthesia, bevacizumab (1.25 mg/ 0.05 mL) was injected intravitreally through the pars plana followed by paracentesis to lower IOP if needed. In eyes after PPV, bevacizumab was injected intracamerally through a paracentesis. An additional intraocular bevacizumab injection was considered to treat the recurrence of rubeosis with IOP elevation.

### Trabeculectomy procedure

The trabeculectomy procedure included the creation of a fornix-based conjunctival flap and a 4x4-mm, half-thickness scleral flap. Small pieces of surgical sponge soaked in 0.4 mg/ ml mitomycin C were then inserted under the conjunctival flap for up to 5 min. The eye was irrigated thoroughly with 200 ml saline. Cataract surgery and/or a three-port PPV were performed at this step as needed. PRP was added during PPV. Trabeculectomy was carried out with a Kelly Descemet’s Membrane Punch (Inami, Tokyo, Japan) followed by peripheral iridectomy. Cauterization was employed to stop active bleeding after iridectomy. The scleral and conjunctival flaps were closed with 10–0 nylon sutures. Postoperatively, argon laser suture lysis and needling revision of the filtration bleb were performed as needed to enhance filtration.

### Postoperative examinations

Postoperative patient examinations were regularly scheduled every day for up to 2 weeks, every 2 weeks for 3 months, monthly up to 6 months and every 3 months thereafter. The IOP was measured by Goldmann applanation tonometry. The mean of two IOP measurements on different days immediately before trabeculectomy were adopted as the preoperative IOP. IOP measurements every 3 months, starting from the 3-month postoperative visit, were used to determine surgical success. IOP measured within a month of the follow-up time points was used. Best-corrected visual acuity (BCVA) was measured using the Landolt C acuity chart at baseline and at the final visit. BCVA was converted into logarithm of the minimal angle of resolution (LogMAR) format (counting fingers, 2.3; hand movements, 2.6; light perception, 3.0; no light perception, 3.6). A marked decrease in BCVA was defined as a >0.3 increase in LogMAR value [9].

### Outcome measures

Surgical failure was defined as insufficient IOP reduction (IOP >21 mmHg, <20% IOP reduction, use of a systemic carbonic anhydrase inhibitor or further glaucoma surgeries), devastating complications (loss of light perception, phthisis bulbi, and endophthalmitis), or significant hypotony (hypotony with IOP ≤5 mmHg continuing ≥6 months and until the last follow-up visit or hypotony that required intervention). Phthisis bulbi was defined as IOP of 0 mmHg or a shrunken eye [16]. Eyes with significant hypotony only after further glaucoma surgeries or cyclodestruction were excluded from the analysis concerning hypotony. Laser suture lysis and bleb needling were not regarded as further glaucoma surgeries. The time of failure was counted as the second consecutive follow-up visit where the IOP criteria were met. Success was classified as qualified or complete according to whether topical IOP-lowering medication were needed or not, respectively.

### Statistical Analyses

Various factors at baseline and postoperatively, surgical complications, and additional interventions were examined. For continuous variables, the Kolmogorov-Smirnov test was used to examine normal distribution and the descriptive statistics were reported accordingly. For comparisons between two groups or BCVA changes, mixed-effects models using clustered robust standard errors by Stata software were used to account for the correlation of both eyes in the same patient. Kaplan-Meier survival analysis was used to examine the cumulative probability of surgical success or hypotony. Among criteria of complete surgical success, significant hypotony was independently analyzed, and complete surgical success excluding hypotony criteria was also examined. Cox proportional hazards regression analysis using clustered robust standard errors by Stata software which accounts for the correlation between fellow eyes was adopted to examine various factors as a potential risk for surgical failure or hypotony. Factors with P-values <0.2 in the univariate Cox regression analysis underwent stepwise multivariate Cox regression analysis. Statistical analysis was performed using SPSS software (IBM SPSS Statistics 20, IBM Corp., New York) and Stata software (version 13.1; StataCorp, TX). For all analyses, a P-value of <0.05 was considered statistically significant.

## Results

### Baseline characteristics

Baseline patient characteristics are shown in Table 1. Two eyes of two patients were excluded because the interval between bevacizumab injection and trabeculectomy was longer than 4 months (23 and 40 months). One eye of one patient was excluded because of a short follow-up period of 4 months due to his death from pancreatic cancer. A total of 61 eyes of 54 patients were therefore included in this study. The most common underlying diagnosis of NVG was proliferative diabetic retinopathy (67.2%) and more than half of the eyes (59.0%) had an extensive peripheral anterior synechia (>50%). Median preoperative IOP was 35.0 mmHg (range, 18.5–58.0). Fifty-eight (95.1%) eyes had intravitreal injections of bevacizumab before trabeculectomy, while three (4.9%) eyes had an intracameral injection. The median interval between intraocular bevacizumab injection and trabeculectomy was 5 days (range, 1–112). The majority of trabeculectomies (90%) were performed by the same surgeon (T.H.), and the remainder were performed by experienced surgeons in our department. PPV and cataract surgery during or after trabeculectomy were performed by a single surgeon (T. H.). Limbus-based conjunctival flaps instead of fornix-based flaps were employed in 3 eyes at the surgeon’s discretion. The average follow-up period after trabeculectomy was 45.0 ± 22.2 months (mean ± standard deviation; range, 12–88).

**Table 1:** Baseline patient characteristics before trabeculectomy.

Number of patients/ eyes		54/ 61
Age (yrs), mean ± SD (range)		59.8±14.5 (31–85)
Sex (female/male)		20/34
Underlying diagnosis of NVG, eyes	Proliferative diabetic retinopathy	41 (67.2%)
	Central retinal vein occlusion	14 (23.0%)
	Ocular ischemic syndrome	3 (4.9%)
	Others	3 (4.9%)
Angle status, eyes	PAS ≤50%	25 (41.0%)
	PAS >50%	36 (59.0%)
Visual acuity, eyes	Finger counting or worse	8 (13.1%)
	0.01–0.1	34 (55.7%)
	0.2–0.5	9 (14.8%)
	0.6–1.0	10 (16.4%)
Preoperative IOP, mmHg, median (range)		35.0 (18.5–58.0)
Number of IOP-lowering medications, median (range)		3 (1–4)
Lens status, eyes	Phakia/ aphakia/ intraocular lens	27 (44.3%)/ 1 (1.6%)/ 33 (54.1%)
Previous pars plana vitrectomy, eyes		19 (31.1%)
Previous trabeculectomy, eyes		1 (1.6%)
Interval between intraocular bevacizumab injections^a^ and trabeculectomy, days, median (range)		5 (1–112)
Follow-up periods after trabeculectomy, months, mean ± SD (range)		45.0 ± 22.2 (12–88)

SD = standard deviation, NVG = neovascular glaucoma, PAS = peripheral anterior synechia, IOP = intraocular pressure.

^a^Fifty-eight (95.1%) and 3 (4.9%) eyes had an intravitreal and intracameral injections of bevacizumab (1.25 mg) before trabeculectomy, respectively.

### IOP outcomes

**Fig 1:**
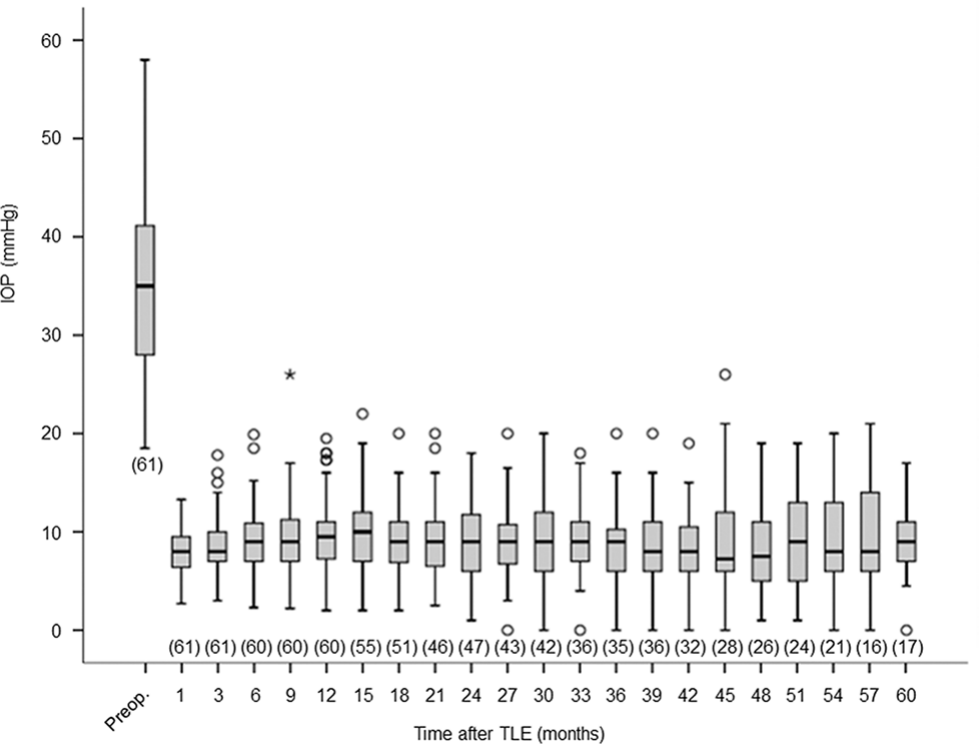
Postoperative intraocular pressure course after trabeculectomy. Box plot of intraocular pressure at each time point. Box, interquartile range; whiskers, non-outlier minimum and maximum; circles, outliers defined as >1.5 times the interquartile range above the 75th percentile or below the 25th percentile; asterisk, extreme value defined as >3 times the interquartile range above the 75th percentile. The number of eyes measured at each time point is shown in parenthesis

**Fig 2:**
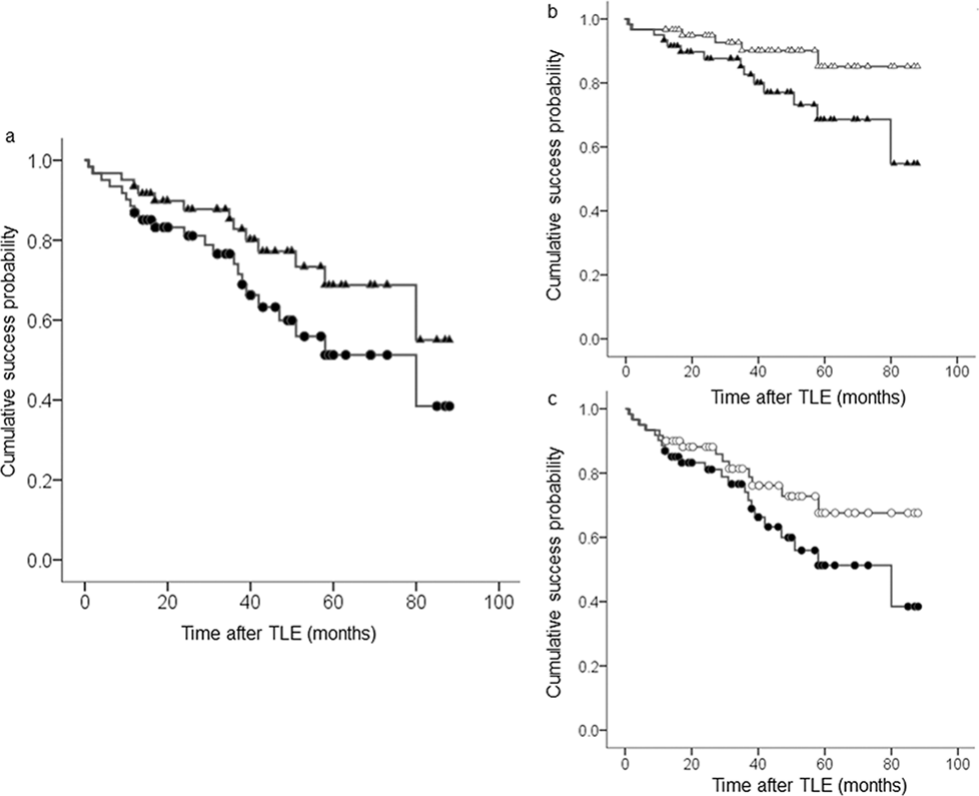
Kaplan-Meier survival analysis of surgical success. **a** Complete success (shaded circle, lower line) vs. qualified success (shaded triangle, upper line). **b** Qualified success, overall criteria (shaded triangle, lower line) vs. exclusion of hypotony criteria (open triangle, upper line). **c** Complete success, overall criteria (shaded circle, lower line) vs. exclusion of hypotony criteria (open circle, upper line).

As shown in Fig 1, IOP decreased to around 10 mmHg throughout the follow-up period. More than half of eyes were followed for at least 42 months after surgery. At the final follow-up visit, median IOP was 8.0 mmHg (range, 2.0–16.0) and the number of IOP-lowering medications was 0.3 ± 0.9. Kaplan-Meier survival analysis showed that complete and qualified surgical success rates were 86.9 ± 4.3% (± standard error) and 93.4 ± 3.2% at 1 year, 74.0 ± 6.1% and 82.8 ± 5.3% at 3 years, and 51.3 ± 8.6% and 68.8 ± 8.0% at 5 years postoperatively (Fig 2A). When excluding hypotony criteria, complete and qualified surgical success rates were 90.2 ± 3.8% and 96.7 ± 2.3% at 1 year, 81.5 ± 5.4% and 90.2 ± 4.3% at 3 years, and 67.7 ± 8.0% and 85.1 ± 6.3% at 5 years postoperatively (Fig 2B and 2C).

### Surgical complications

Surgical complications and additional interventions are shown in Table 2. No major intraoperative complications such as marked hyphema were encountered. Relatively frequent early postoperative complications were hyphema with niveau formation, choroidal detachment, fibrin formation and bleb leak. The most frequent late complication was vitreous hemorrhage, which occurred in one fourth of eyes and was treated by PPV. Hypotony maculopathy, bullous keratopathy and phthisis bulbi occurred in three eyes, two eyes and one eye, respectively, and these complications developed only in eyes with significant hypotony. No bleb infections developed. One third of eyes required PPV after a mean of 12 months. One half of phakic eyes needed cataract surgery after an average of 25 months. Bleb needling was performed in 10 eyes and two of these eyes required bleb revision or a second trabeculectomy. An additional intraocular bevacizumab injection was needed in 5 eyes to treat the recurrence of rubeosis. Of these, three eyes underwent subsequent vitrectomy for vitreous hemorrhage (two eyes) or macular edema (one eye). Bleb revision and second trabeculectomy were performed in three eyes and one eye, respectively.

**Table 2:** Surgical complications and additional intervention.

Items	Eyes (%)	Remarks
<Intraoperative complications>		
marked hyphema (pupil not visible)	0 (0%)	
<Early complications^a^>		
Postoperative hyphema with niveau formation	20 (32.8%)	Duration, median (range): 5 (1–39) days.
Choroidal detachment	10 (16.4%)	Duration, median (range): 6 (1–58) days.
Fibrin formation	10 (16.4%)	
Bleb leak	8 (13.1%)	All occurred ≤2 weeks postop. Treated by conjunctival sutures in 7 (87.5%) eyes.
Shallow anterior chamber	2 (3.3%)	Treated by transconjunctival scleral flap sutures 8 days postop. in 1 eye.
Malignant glaucoma	1 (1.6%)	Treated by PPV+cataract surgery 9 days postop.
<Late complications^b^>		
Vitreous hemorrhage	15 (24.6%)	Treated by PPV at 12.0 ±8.7 (0.2–27) months.
Hypotony maculopathy	3 (4.9%)	Treated by transconjunctival scleral flap sutures at 13 months in 1 eye, or intrableb injection of autologous blood at 36 months in 1 eye.
Bullous keratopathy	2 (3.3%)	Occurred at 19, 76 months.
Phthisis bulbi	1 (1.6%)	Occurred at 27 months.
Bleb leak	0 (0%)	
Choroidal detachment	0 (0%)	
Bleb infection	0 (0%)	
<Additional intervention>		
PPV	20 (32.8%)	Performed 12.4 ± 8.7 (0.2–27) months postop. Indications: Vitreous hemorrhage in 15 (75%) eyes, epiretinal membrane in 2 (10%) eyes, cystoid macular edema in 2 (10%) eyes, malignant glaucoma in 1 (5%) eye.
Cataract surgery^c^	10 (52.6%)	Performed 24.9 ± 19.9 (6–59) months postop.
Bleb needling	10 (16.4%)	Performed 9.2 ± 10.9 (1.5–34) months postop.
Intraocular bevacizumab injections	5 (8.2%)	All due to recurrence of rubeosis. Number of injections: 1 (3 eyes), 3 (2 eyes).
Bleb revision	3 (4.9%)	Performed 1, 2, 17 months postop.
Trabeculectomy	1 (1.6%)	Performed 13 months postop.

PPV = pars plana vitrectomy.

^a^Occured within 3 months post op.

^b^Occured after 3 months post op.

^c^Nineteen phakic eyes after trabeculectomy were analyzed.

### Visual acuity

With respect to visual acuity, LogMAR values of BCVA at baseline and the final follow-up visit were 1.20 ± 0.78 and 0.98 ± 0.81, respectively. The mean BCVA was significantly improved after trabeculectomy (P = 0.014). A marked deterioration in BCVA, defined as an increase in LogMAR >0.3, occurred in 7 eyes; 4 in non-hypotonic eyes and 3 in hypotonic eyes. BCVA deterioration was significantly more frequent in eyes with significant hypotony, (p = 0.006).

### Complete surgical success

Twenty-two eyes did not meet the criteria for complete success: 10 eyes required topical IOP-lowering medications 23.0 ± 15.3 months (4–47) after surgery, two eyes underwent bleb revision one or two months after surgery, one eye lost light perception at 58 months, and nine eyes developed significant hypotony 34.0 ± 22.8 months (9–80) after surgery. Among the 10 eyes that received additional topical medications, one eye underwent bleb revision 11 months later, and one eye received systemic carbonic anhydrase inhibitor four months later. One hypotonic eye was regarded as phthisis bulbi 18 months later.

Comparisons of factors between eyes with (n = 39) and without (n = 22) complete success are shown in Table 3. Lower preoperative IOP (≤30 mmHg) and PPV after trabeculectomy were significantly more frequent in failed eyes than in successful eyes (P = 0.024, 0.041, respectively). The follow-up period after trabeculectomy was significantly shorter in successful eyes than failed eyes (P = 0.038). In Table 4, the multivariate Cox regression analysis showed that lower preoperative IOP (≤30 mmHg) and PPV after trabeculectomy were significant risk factors for surgical failure [hazard ratio (HR), 2.92, 2.32; 95% confidence interval (CI), 1.21 to 7.09, 1.02 to 5.28; P = 0.018, 0.045, respectively.

**Table 3:** Comparisons of various factors between eyes with and without complete surgical success.

Factors	Total eyes (61 eyes, 100%)	Success (39 eyes, 63.9%)	Failure (22 eyes, 36.1%)	P value^a^
Age (yrs, mean ± standard deviation)	59.8±14.5	60.4 ± 13.9	58.8 ± 15.7	0.71
Age (≤50 yrs)	16 (26.2%)	10 (25.6%)	6 (27.3%)	0.90
Sex (male)	42 (68.9%)	26 (66.7%)	16 (72.7%)	0.64
Predisposing diagnosis (PDR)	41 (67.2%)	26 (66.7%)	15 (68.2%)	0.91
Preoperative IOP (mmHg, median, range)	35.0 (18.5–58.0)	36.5 (22.0–55.5)	28.8 (18.5–58.0)	0.23
Preoperative IOP (≤30 mmHg)	24 (39.3%)	11 (28.2%)	13 (59.1%)	0.024
NVG stage (closed angle)	36 (59.0%)	25 (64.1%)	11 (50.0%)	0.29
Preoperative lens status (intraocular lens, aphakia)	34 (55.7%)	23 (59.0%)	11 (50.0%)	0.50
Previous PPV	19 (31.1%)	12 (30.8%)	7 (31.8%)	0.93
Interval between intraocular bevacizumab injections and TLE (days, median, range)	5 (1–112)	4 (1–83)	6.5 (1–112)	0.44
Interval between intraocular bevacizumab and TLE (≤4 days)	28 (45.9%)	21 (53.8%)	7 (31.8%)	0.09
TLE combined with PPV	7 (11.5%)	4 (10.3%)	3 (13.6%)	0.72
Postoperative lens status (intraocular lens, aphakia)	42 (68.9%)	27 (69.2%)	15 (68.2%)	0.93
Postoperative vitreous status (avitreous)	27 (44.3%)	17 (43.6%)	10 (45.5%)	0.88
Postoperative hyphema	20 (32.8%)	13 (33.3%)	7 (31.8%)	0.90
Postoperative choroidal detachment	10 (16.4%)	5 (12.8%)	5 (22.7%)	0.32
Postoperative fibrin formation	10 (16.4%)	5 (12.8%)	5 (22.7%)	0.37
Postoperative bleb leak	8 (13.1%)	3 (7.7%)	5 (22.7%)	0.14
Cataract surgery after TLE^b^	10 (52.6%)	8 (8/12, 66.7%)	2 (2/7, 28.6%)	0.12
PPV after TLE	20 (32.8%)	9 (23.1%)	11 (50.0%)	0.041
Follow-up periods after TLE (months, mean ± standard deviation)	45.0 ± 22.2	40.7 ± 22.7	52.6 ± 19.6	0.038

PDR = proliferative diabetic retinopathy, IOP = intraocular pressure, NVG = neovascular glaucoma, PPV = pars plana vitrectomy, TLE = trabeculectomy

^a^Mixed-effects models with clustered robust standard errors were used to account for the correlation of both eyes in the same patient.

^b^Nineteen phakic eyes after trabeculectomy were analyzed.

**Table 4:** Cox regression analysis of various factors as a risk for surgical failure by complete success criteria.^a^

		Univariate Cox^b^			Multivariate Cox^b^ ^,^ ^c^		
Factors	No. of eyes in failed eyes (%)	Hazard ratio	95% CI	P value	Hazard ratio	95% CI	P value
Age (yrs)	NA	1.00	0.96–1.03	0.90			
Age (≤50 yrs)	6 (27.3)	1.10	0.39–3.08	0.86			
Sex (male)	16 (72.7)	1.47	0.54–4.01	0.46			
Predisposing diagnosis (PDR)	15 (68.2)	0.84	0.33–2.12	0.71			
Preoperative IOP (mmHg)	NA	0.97	0.91–1.03	0.28			
Preoperative IOP (≤30 mmHg)	13 (59.1)	2.70	1.12–6.49	0.027	2.92	1.21–7.09	0.018
NVG stage (closed angle)	11 (50.0)	0.52	0.23–1.15	0.11			
Preoperative lens status (intraocular lens, aphakia)	11 (50.0)	0.87	0.39–1.95	0.74			
Previous PPV	7 (31.8)	1.47	0.65–3.32	0.36			
Interval between intraocular bevacizumab and TLE (days)	NA	1.01	1.00–1.02	0.07			
Interval between intraocular bevacizumab and TLE (≤4 days)	7 (31.8)	0.44	0.18–1.07	0.07			
TLE combined with PPV	3 (13.6)	1.02	0.26–3.96	0.98			
Postoperative lens status (intraocular lens, aphakia)	15 (68.2)	1.14	0.48–2.73	0.76			
Postoperative vitreous status (avitreous)	10 (45.5)	1.35	0.61–3.02	0.46			
Postoperative hyphema	7 (31.8)	0.77	0.34–1.78	0.54			
Postoperative choroidal detachment	5 (22.7)	1.49	0.52–4.27	0.46			
Postoperative fibrin formation	5 (22.7)	1.58	0.55–4.50	0.39			
Postoperative bleb leak	5 (22.7)	1.72	0.55–5.39	0.35			
Cataract surgery after TLE^d^	2 (28.6)	0.35	0.07–1.66	0.18			
PPV after TLE	11 (50.0)	2.12	0.90–4.98	0.09	2.32	1.02–5.28	0.045

CI = confidence interval, NA = not applicable, PDR = proliferative diabetic retinopathy, IOP = intraocular pressure, NVG = neovascular glaucoma, PPV = pars plana vitrectomy, TLE = trabeculectomy.

^a^Twenty-two eyes failed to meet the complete success criteria.

^b^Adjusted to account for the correlation of both eyes in the same patient.

^c^Variables with P <0.2 in the univariate Cox regression analysis were entered into the stepwise multivariate analysis.

^d^Nineteen phakic eyes after TLE were analyzed.

### Complete surgical success excluding hypotony criteria

Complete surgical success excluding hypotony criteria was also evaluated. Nine eyes met the failure criteria of hypotony. Of these, one eye developed phthisis bulbi and therefore was regarded as failure even excluding hypotony criteria. In comparisons of factors between eyes with (n = 47) and without (n = 14) the success criteria, PPV after trabeculectomy was significantly less in successful eyes than failed eyes (p = 0.043). By multivariate Cox regression analysis, postoperative avitreous status and PPV after trabeculectomy were identified as significant risk factors for surgical failure (HR, 3.70, 4.06: 95% CI, 1.16 to 11.8, 1.30 to 12.7; P = 0.028, 0.016, respectively).

### Significant hypotony

Significant hypotony developed in ten eyes, but one eye was excluded from the analysis because persistent hypotony occurred after a second trabeculectomy. Only three of nine eyes with significant hypotony were free from major complications such as hypotony maculopathy, bullous keratopathy, phthisis bulbi, and marked VA loss. When comparing factors between eyes with (n = 9) and without (n = 51) significant hypotony, lower preoperative IOP (≤30 mmHg) and postoperative bleb leak were significantly more frequent in eyes with significant hypotony than in those without (P = 0.023, 0.048, respectively). Furthermore, lower preoperative IOP (≤30 mmHg) was identified as a significant risk factor for hypotony by multivariate Cox regression analysis (HR, 6.64: 95% CI, 1.47 to 30.0; P = 0.014).

## Discussion

Effects of adjunctive use of intraocular anti-VEGF agents on glaucoma filtration surgeries for NVG have been evaluated in a number of studies. Less postoperative hemorrhagic complications and better surgical outcomes were anticipated because of the remarkable rapid and steady suppression of rubeosis after an intraocular injection of bevacizumab [17–19]. Indeed, postoperative hyphema was significantly less frequent when bevacizumab was used prior to trabeculectomy [9,11] or tube shunt surgery [20–22], although a statistically significant difference vs. control group was not achieved in other reports [6,10,12,23]. Furthermore, short-term surgical success rate was significantly better in eyes with preoperative bevacizumab injections in trabeculectomy [9,12] and tube shunt surgery [20,22]. However, negative results were also reported for trabeculectomy [6,10,11] and tube shunt surgery [21,23]. The inconsistent results may stem from differences in the definition of surgical success, patient backgrounds and surgical procedures among different studies. The small sample size (≤30 eyes) and short follow-up periods (<20 months) of these studies may also influence the results. In this study, we examined 61 NVG eyes with a mean follow-up period of 45 months to address the long-term outcome of trabeculectomy with adjunctive use of intraocular bevacizumab.

Our surgical success rate was favorably compared with previous reports on the long-term outcomes of filtration surgeries for NVG (Table 5). Surgical success rate of trabeculectomy at 1 year was about 65 to 85% with preoperative bevacizumab treatment and 30 to 75% without treatment [2–4,6,11,24–27]. Similarly, the success rate of tube shunt surgery at 1 year was 55 to 95% with preoperative bevacizumab treatment and 25 to 75% without treatment [20,22,23,28–34]. Of note, success rates without bevacizumab use dropped to a level of <70% at ≥2 years in both trabeculectomy [2–4,6,26] and tube shunt surgeries [29,31–34], while recent reports on filtration surgeries with adjunctive bevacizumab demonstrated >70% of success rates up to 3 years postoperatively, which was equivalent to our study [25,28]. Netland et al. reported that the surgical success of Ahmed Glaucoma Valve surgery without bevacizumab use in NVG eyes was significantly worse than in non-NVG eyes [31]. Our results up to 5 years were comparable with those of recent large-scale studies on non-NVG eyes [35–37]. Although direct comparison of surgical success rate between different studies may be hampered by heterogeneity of patient backgrounds and success criteria, adjunctive use of bevacizumab may have the potential to promote long-term surgical success of trabeculectomy in NVG eyes.

**Table 5:** Cumulative probability of surgical success at 1 year or later in neovascular glaucoma.

				Cumulative probability of surgical success (%) with the upper limits of intraocular pressure (≤20–21 mmHg)				
Type of filtration surgeries	Preop. anti-VEGF agents	Hypotony criteria for surgical failure	No. of eyes	@ 1 year	@ 2 years	@ 3 years	@ 5 years	Studies
TLE	+	-	61	90.2c, 96.7q		81.5c, 90.2q	67.7c, 85.1q	Current study
TLE	+	-	24	65.2q				Takihara et al. 2011^11^
TLE	+	-	15	73q				Miki et al. 2011^24^
TLE	+	-	12	83.3q		83.3q		Kobayashi et al. 2015^25^
TLE	-	-	101	62.6q	58.2q		51.7q	Takihara et al. 2009^4^
TLE	-	-	35	67q	62q	62q		Kiuchi et al. 2006^3^
TLE	-	-	15	71.4c	62.5c	52.1c		Mandel et al. 2002^26^
TLE	-	-	34	71q	67q	61q	28q	Tsai JC 1995^2^
TLE	+	+	61	86.9c, 93.4q		74.0c, 82.8q	51.3c, 68.8q	Current study
TLE	-	+	30	74.7q	57.6q			Al-Obeidan et al. 2008^5^
TLE	-	+	24	29q				Hyung et al. 2001^27^
TLE^a^	-	+	105	86.1q	71.8q	69.3q	53.1q	Gedde et al. 2009,^35^ 2012^36^
TLE^a^	-	+	428		80c, 87q			Kirwan et al. 2014^37^
A	+	+	20	75c, 95q				Mahdy et al. 2013^22^
A	+	+	25	84.0c, q				Zhou et al. 2013^20^
A	+	+	19	53c, 79q				Sevim et al. 2013^23^
A	+	+	35	82.9q	74.1q	71.0q		Zhang et al. 2014^28^
A	-	-	19	68.8q			42.9q	Kim et al. 2009^29^
A	-	-	27	64.1c, 76.0q				Teixeira et al. 2011^30^
A	-	+	38	73.1q	61.9q	20.6q		Netland et al. 2010^31^
A	-	+	20	25c, 50q				Mahdy et al. 2013^22^
A	-	+	28	64.3c, 75.0q				Zhou et al. 2013^20^
A	-	+	22	28c, 64q				Sevim et al. 2013^23^
A	-	+	33	61.3c	59.3c			Yildirim et al. 2009^32^
A	-	+	38	63.2c	56.1c	43.2c	25.2c	Yalvac et al. 2007^33^
M	-	+	27	37c	29.6c	29.6c	29.6c	Yalvac et al. 2007^33^
M	-	+	145	72q	62q		40q	Every et al. 2006^34^
B^a^	-	+	107	96.2q	88.8q	84.9q	70.2q	Gedde et al. 2009,^35^ 2012^36^

VEGF = vascular endothelial growth factor, TLE = trabeculectomy, A = Ahmed glaucoma valve, B = Baerveldt glaucoma implant, M = Molteno implant, c = complete success (no medication), q = qualified success (medication allowed).

^a^Non-neovascular glaucoma eyes.

Surgical success in this study was defined as a sufficient IOP reduction, no serious complications and no significant hypotony. Hypotony criteria may not be an appropriate indicator for surgical failure since patients with postoperative IOP in hypotony ranges are often visually asymptomatic [38]. However, the guidelines of the World Glaucoma Association recommend setting a lower IOP limit (i.e. 6 mmHg) for IOP success [13]. As shown in Table 5, previous studies on filtration surgeries in NVG eyes have not necessarily defined a lower IOP limit and the influence of hypotony criteria on surgical success in NVG eyes has never been addressed. In our study, significant hypotony developed in 15% of cases after a mean follow-up of 34 months, which increased the postoperative failure rate steadily over time from 3.3%, to 7.4% and 16.3% at 1, 3, and 5 years, respectively. Late-onset hypotony after trabeculectomy is not a rare complication. Bindlish et al. reported that delayed hypotony (IOP <6 mmHg at any time point from 6 months to 5 years) occurred in 42% of 123 non-NVG eyes, which underwent primary trabeculectomy with mitomycin C, after a mean follow-up of 26 months [39]. The hypotony reduced the success rate from 83.0% to 57.7% at year 5. Furthermore, half of cases with marked VA deterioration had delayed hypotony. A significant association between late-onset hypotony and VA deterioration was confirmed in our study. In the “Tube Versus Trabeculectomy Study” on non-NVG eyes with previous glaucoma and/or cataract surgeries, persistent hypotony (IOP ≤5 mmHg on two consecutive follow-up visits after 3 months) occurred in 12.4% of 105 eyes which underwent trabeculectomy. Hypotony accounted for 30% of surgical failures of trabeculectomy at 5 years [36]. Kirwan et al. reported that 7.2% of 428 non-NVG cases, which underwent primary trabeculectomy mostly using anti-fibrotics, developed late-onset hypotony (IOP <6 mmHg between 6 months and the final follow-up visit) with a median follow-up of 40 months [37]. Although the incidence of delayed hypotony varies among the studies, possibly due to differences in the definition of hypotony, surgical procedures, and patient’s backgrounds, it may significantly affect the long-term outcome of trabeculectomy. Furthermore, given that majority of our cases with significant hypotony had major complications such as hypotony maculopathy, bullous keratopathy, phthisis bulbi, and marked VA loss, inclusion of lower limit of IOP in the success criteria of trabeculectomy may be appropriate.

Two different risk factors, lower baseline IOP (<30 mmHg) and PPV after trabeculectomy, were identified by multivariate Cox regression analysis for surgical failure by complete success criteria. Lower baseline IOP was also a risk factor for significant hypotony. A low baseline IOP may indicate compromised aqueous production due to underlying ischemic pathology in NVG eyes given that baseline IOP level was not proportional to the degree of angle closure. Therefore, eyes with low aqueous production may have relatively low IOP at baseline and may develop late-onset hypotony due to successful filtration. In contrast, PPV after trabeculectomy was identified as the risk of surgical failure regardless of the inclusion of hypotony criteria. Postoperative avitreous status, which includes both previous PPV and combined PPV with trabeculectomy, was also a risk factor for surgical failure excluding hypotony criteria, i.e. insufficient IOP reduction. Previous PPV is a risk factor for surgical failure of trabeculectomy without bevacizumab use in NVG eyes [3,4]. An additional PPV was a newly identified risk factor for surgical failure after trabeculectomy in NVG eyes. PPV after trabeculectomy was performed in 20 eyes, most of which were due to vitreous hemorrhage, after a mean of 12 months in our study. Of these, 17 eyes had no recurrence of anterior segment neovascularization, indicating that vitreous hemorrhage may have been caused by the evolution of the posterior vitreous detachment rather than the exacerbation of the retinal ischemia in most cases. PPV was performed transconjunctivally with either a 23 or a 25 gauge instrument and caused no major intraoperative and postoperative complications. Recently, Kunikata et al. reported that 27% of 15 eyes lost IOP control after 25-gauge PPV in eyes with a filtering bleb after trabeculectomy [40]. Therefore, PPV may be a risk factor for bleb failure in NVG eyes even when neovascularization has subsided and a transconjunctival small-gauge system is employed. Tube shunt surgeries may be more favorable for NVG eyes with compromised aqueous production or subsequent PPV although it is currently unknown if these risk factors are also applicable to tube shunt surgeries. Further studies are warranted concerning the comparison of risk factors for surgical failure in NVG eyes between trabeculectomy and tube shunt surgeries.

This study has several limitations including the retrospective design and the lack of a control group. We followed up every NVG case as long as possible with a solid protocol of treatment using adjunctive intraocular bevacizumab injections [14] and follow-up examinations to reduce potential bias. Consequently, only one case was excluded due to drop out before 12 months of follow-up. Our group has previously reported that preoperative intravitreal injection of bevacizumab significantly reduced postoperative hyphema and improved the short-term surgical success of trabeculectomy in a retrospective comparative study [9]. Therefore, we examined the long-term surgical outcomes in a relatively large number of consecutive cases including initial cases, compared the success rate with other studies, and explored the risk factors of surgical failure. However, as pointed out by a recent systematic review [41], clear evidence of the effectiveness of anti-VEGF agents for NVG treatment is still insufficient. Our results may facilitate design of a prospective study to validate the effectiveness of this treatment strategy for NVG.

In conclusion, the long-term outcome of trabeculectomy following intraocular bevacizumab injections for NVG was favorable. Among risk factors identified for surgical failure by complete success criteria, lower baseline IOP was associated with the development of significant hypotony, while additional PPV was related to the insufficient IOP reduction. A long-term careful follow-up is mandatory for NVG eyes treated by trabeculectomy with adjunctive bevacizumab injections.
